# Exploring the Branching Pattern of the Posterior Division of the Internal Iliac Artery: An Analysis Based on 75 Computed Tomography Angiographies

**DOI:** 10.1007/s00192-024-05944-8

**Published:** 2024-10-09

**Authors:** Paweł Hajdyła, Patryk Ostrowski, Michał Bonczar, Jakub Gliwa, Ameen Nasser, Kyrylo Shafarenko, Wadim Wojciechowski, Jerzy Walocha, Mateusz Koziej

**Affiliations:** 1 Department of Anatomy, Jagiellonian University Medical College, Mikołaja Kopernika 12, 33-332 Kraków, Poland; 2 Youthoria, Youth Research Organization, Kraków, Poland; 3 Department of Radiology, Jagiellonian University Medical College, Kraków, Poland

**Keywords:** Internal iliac artery, Anatomy, Pelvis, Surgery, Variation

## Abstract

**Introduction and Hypothesis:**

The internal iliac artery stands as the main blood supplier of the pelvis, serving as the primary source of blood for the pelvic viscera while also nourishing the musculoskeletal framework within. The arterial anatomy of the pelvis exhibits a vast array of variations, especially regarding the branching pattern of the internal iliac arteries. The posterior division of the internal iliac artery (PDIIA) may also have variable topography, especially regarding the location of its origin in the pelvic region.

**Methods:**

A retrospective study was carried out to determine the anatomical variations, prevalence, and morphometric data of the PDIIA and its branches. A total of 75 computed tomography angiographies were analyzed.

**Results:**

The most prevalent branch of the PDIIA was the superior gluteal artery, as it was present in 114 of the studied cases (77.03%). The median diameter of the PDIIA at its origin was 6.66 mm. The median cross-sectional area of the PDIIA at its origin was set to be 34.59 mm^2^.

**Conclusion:**

Our study highlights the critical significance of understanding the PDIIA and its branches in surgical interventions aimed at managing pelvic hemorrhage. The present study provides valuable insights into the precise localization and characteristics of the PDIIA and its branches, which are essential for surgical procedures targeting specific vessels to control bleeding effectively. Owing to the high level of variability of the branching pattern of the PDIIA, a novel classification system consisting of six types was created.

## Introduction

The internal iliac artery stands as the main blood supplier of the pelvis, serving as the primary source of blood for the pelvic viscera while also nourishing the musculoskeletal framework within. Notably, it extends its reach to the gluteal region, medial thigh zones, and the perineum through its intricate network of branches. Following the division of the internal iliac artery into anterior and posterior branches, the posterior division typically gives origin to three distinct parietal arteries, mainly the iliolumbar artery, the lateral sacral artery, and the superior gluteal artery [1].

The arterial anatomy of the pelvis exhibits a vast array of variations, especially regarding the branching pattern of the internal iliac arteries. The posterior division of the internal iliac artery (PDIIA) may also have variable topography, especially regarding the location of its origin in the pelvic region. Rarely, the PDIIA may not even be present, with its branches originating from other nearby arteries [2]. Although numerous studies have been conducted on the overall branching pattern of the internal iliac artery, there is a deficit of studies focusing on the anatomy of the PDIIA. Having adequate knowledge regarding the topographical and morphometric properties of the PDIIA is paramount for physicians performing pelvic surgeries and endovascular procedures [3]. Knowledge regarding the branching pattern of the PDIIA may be essential in clinical cases where pelvic hemorrhage needs to be effectively controlled [3]. Hence, the present study is aimed at showcasing the topography of the PDIIA and its branches using novel arterial anatomical maps and efficient ways of localizing each branch. This can aid in more efficient localization of the origin of the PDIIA and its branches, subsequently decreasing the risk of iatrogenic injury to these vessels when performing various pelvic surgeries, as well as effectively controlling hemorrhage in the pelvic region.

## Materials and Methods

### Approvement of the Bioethical Committee

The research protocol was submitted for evaluation and approved by the Jagiellonian University Bioethical Committee, Cracow, Poland (1072.6120.254.2022). The research was conducted in accordance with the permitted criteria throughout the subsequent phases.

### Study Group

A retrospective study was carried out to determine the anatomical variations, prevalence, and morphometric data of the PDIIA and its branches. The study analyzed the results from 75 consecutive patients who had pelvic computed tomography angiography (CTA) at the Department of Radiology, Jagiellonian University Medical College, Cracow, Poland, between 2017 and 2022. Each patient's results were examined bilaterally and were analyzed at the Department of Anatomy of the Jagiellonian University Medical College in August 2022. Exclusion criteria were set as follows:

1. Pelvic or abdominal trauma affecting the course of the PDIIA and/or its branches.
2. Significant artifacts that prevented accurate and precise imaging and/or measurement of the PDIIA and/or its branches.
3. Low-quality and illegible images
4. Significant lack of filling the whole arterial system with contrast material.

Defects meeting the exclusion criteria that affected only one side of the CTA without impacting the opposite side did not disqualify the entire CTA but only the side with the defect. Only two PDIIAs were excluded owing to significant artifacts. Finally, 148 PDIIAs of 73 patients met the required criteria.

### Acquisition of Results

All pelvic CTA were performed on a 128-slice scanner CT (Philips Ingenuity CT; Philips Healthcare). The primary CTA imaging parameters were as follows: collimation/increment: 0.625/0.3 mm; tube current: 120 mAs; field of view: 210 mm; matrix size: 512 × 512.

All of the patients received intravenous administration of contrast material at a dose of 1 ml/kg (standard dose). A non-ionic contrast medium (CM) containing 350 mg of iodine per milliliter was used (Jowersol 741 mg/ml; Optiray®; Guerbet, Villepinte, France). CT data acquisition was triggered using a real-time bolus-tracking technique (Philips Healthcare) with the region of interest (ROI) placed in the ascending aorta. The CM was intravenously injected using a power injector at a flow rate of 5 ml/s. This was immediately followed by the injection of 40 ml of saline solution at the same flow rate. Following the injection of CM and saline, image acquisition was automatically started with a 2-s delay when the attenuation trigger value reached a threshold of 120 Hounsfield units (HU). Scanning was performed in the caudocranial direction.

The CTAs were analyzed on a dedicated workstation at the Department of Anatomy of the Jagiellonian University Medical College. Materialize Mimics Medical version 21.0 software (Materialise NV, Leuven, Belgium) was used to ensure the highest possible quality of the visualizations and measurements and minimize potential bias. Three-dimensional (3D) reconstructions of each scan were developed, employing a set of settings, severally adjusted to each scan. A volume rendering opacity range oscillated from 25 to 80 HU for the lower limit and up to 3,070 HU for a higher limit. The range was individually adjusted to each PDIIA after a visual investigation.

### Evaluation and Measurements

At the start of each evaluation, the authors ensured that each PDIIA, its branches, and the surrounding anatomical area were completely visualized. Subsequently, each branch of the PDIIA was identified by following its course. The origin of the PDIIA and its branches were assessed and descriptively recorded. Furthermore, each PDIIA origin and branching pattern has been descriptively noted to establish the variation types in further stages. Measurements of each PDIIA were then performed by two independent researchers, and an average was calculated from their results. All measurements were rounded to two decimal places. The following measurements were taken:

1. PDIIA diameter at its origin (mm)
2. PDIIA cross-sectional area at its origin (mm^2^)
3. PDIIA angle of departure (°)
4. Number of branches departing from the PDIIA
5. Distance from the origin of the internal iliac artery to the bifurcation into posterior and anterior division (mm)
6. Iliolumbar artery diameter at its origin (mm)
7. Iliolumbar artery cross-sectional area at its origin (mm^2^)
8. Iliolumbar artery angle of departure (°)
9. Lateral sacral artery diameter at its origin (mm)
10. Lateral sacral artery cross-sectional area at its origin (mm^2^)
11. Lateral sacral artery angle of departure (°)
12. Superior gluteal artery diameter at its origin (mm)
13. Superior gluteal artery cross-sectional area at its origin (mm^2^)
14. Superior gluteal artery angle of departure (°)
15. Accessory iliolumbar artery diameter at its origin (mm)
16. Accessory iliolumbar artery cross-sectional area at its origin (mm^2^)
17. Accessory iliolumbar artery angle of departure (°)
18. Accessory lateral sacral artery diameter at its origin (mm)
19. Accessory lateral sacral artery cross-sectional area at its origin (mm^2^)
20. Accessory lateral sacral artery angle of departure (°)
21. Distance between the origin of the PDIIA and the origin of the iliolumbar artery (mm)
22. Distance between the origin of the PDIIA and the origin of the lateral sacral artery (mm)
23. Distance between the origin of the PDIIA and the origin of the superior gluteal artery (mm)
24. Distance between the origin of the iliolumbar artery and the origin of the lateral sacral artery (mm)
25. Distance between the origin of the iliolumbar artery and the origin of the superior gluteal artery (mm)
26. Distance between the origin of the lateral sacral artery and the origin of the superior gluteal artery (mm)
27. Distance between the PDIIA and the anterior superior iliac spine (straight line; mm)
28. Angle between the section designated by the anterior superior iliac spines and the section designated by the anterior superior iliac spine and the origin of the PDIIA (°)

**Fig. 1:**
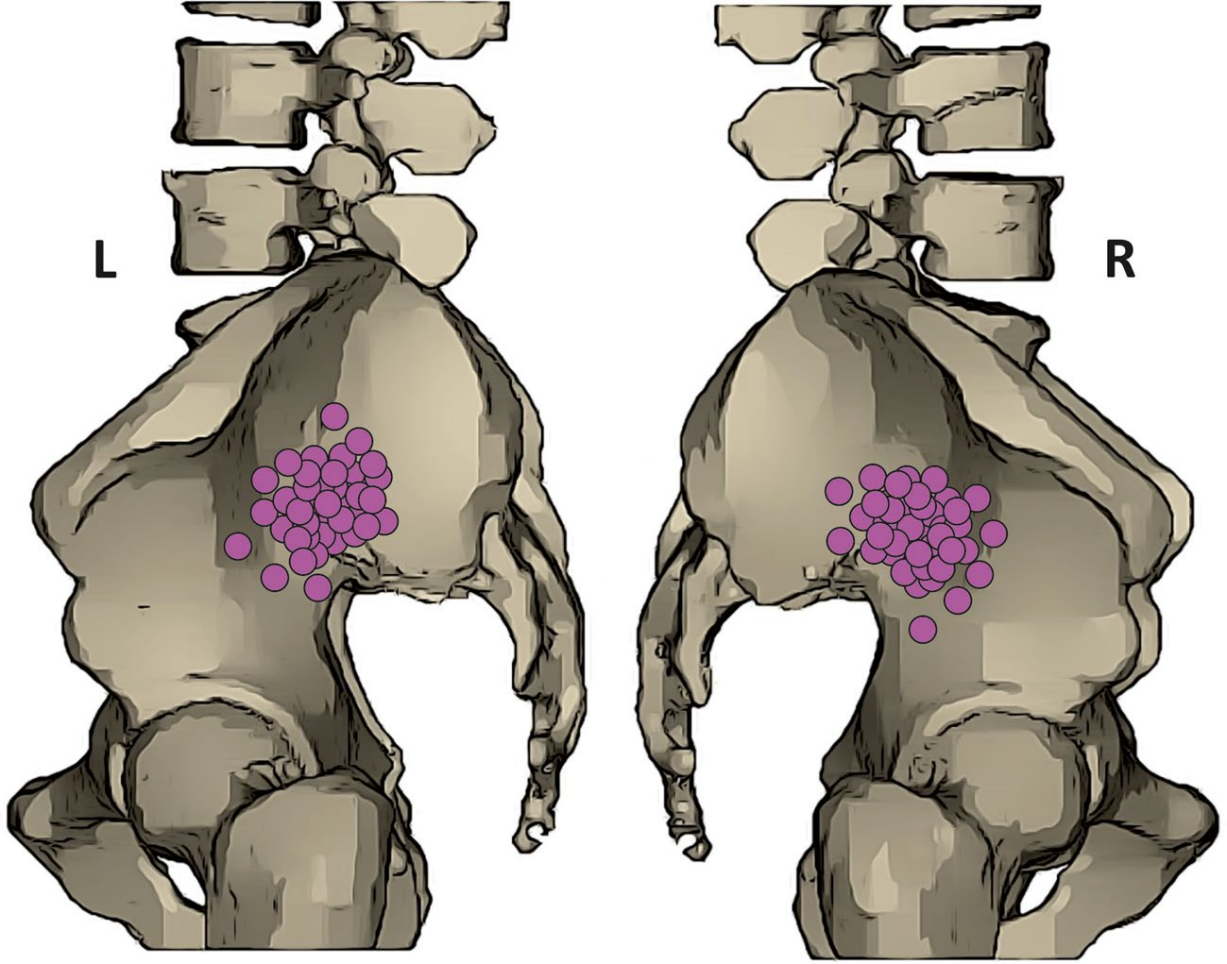
Anatomical map presenting the occurrence of the posterior division of the internal iliac artery from the lateral view

Furthermore, a set of measurements has been taken to create an anatomical map of the occurrence of the origin of the PDIIA in the lateral view (Fig. 1).

### Statistical Analysis

Statistical analysis was performed with SATISTICA v13.1 (StatSoft Inc., Tulsa, OK, USA). The frequencies and percentages presented qualitative features. The Shapiro–Wilk test was used to assess the normal distribution. Quantitative characteristics were presented by medians and upper and lower quartiles (UQ, LQ), as well as means and standard deviation (SD), depending on the verified normality of the data. Statistical significance was defined as *p* < 0.05. Mann–Whitney *U* and Wilcoxon signed-rank tests were used to establish potential differences between groups. Spearman's rank correlation coefficient was used to determine possible correlations between the parameters.

## Results

### Qualitative Results

All following results are presented with respect to the number of PDIIA rather than the number of patients. A total of 148 PDIIA were analyzed, of which 85 (57.4%) were from females, whereas 63 (42.6%) were from males. The left and right sides were analyzed in equal amounts. Patients’ mean age was 52.0 (SD = 15.23; minimum = 22.0; maximum = 86.0). The most prevalent branch of the PDIIA was the superior gluteal artery, as it was present in 114 of the studied cases (77.03%). The most common first branch of the PDIIA was found to be the iliolumbar artery (57.76%). In contrast, the most common last branch of the PDIIA was found to be the superior gluteal artery (90.52%). In two cases, the accessory iliolumbar artery originated from the PDIIA. The accessory lateral sacral artery originated from the PDIIA in the other two cases. Detailed qualitative results are presented in Table 1.

**Table 1:** Qualitative results of the data analysis

Category	*N*	Percentage
Patients’ sex		
Female	85	57.4
Male	63	42.6
Patients’ side		
Left	74	50.0
Right	74	50.0
Number of sides in which the mentioned arteries originated directly from the posterior division of the internal iliac artery		
Superior gluteal artery	114	77.03
Iliolumbar artery	79	53.38
Lateral sacral artery	48	32.43
Obturator artery	12	8.11
Inferior gluteal artery	8	5.41
Accessory lateral sacral artery	3	2.03
Accessory iliolumbar artery	2	1.35
Number of sides in which the mentioned artery was the first branch of the posterior division of the internal iliac artery		
Iliolumbar artery	67	57.76
Lateral sacral artery	28	24.14
Obturator artery	10	8.62
Superior gluteal artery	8	6.90
Inferior gluteal artery	3	2.59
Number of sides in which the mentioned artery was the last branch of the posterior division of the internal iliac artery		
Superior gluteal artery	105	90.52
Lateral sacral artery	7	6.03
Inferior gluteal artery	4	3.45
Other		
Number of sides in which at least one branch of the anterior division of the internal iliac artery originated from the posterior division of the internal iliac artery	20	13.51

### Variations

**Fig. 2:**
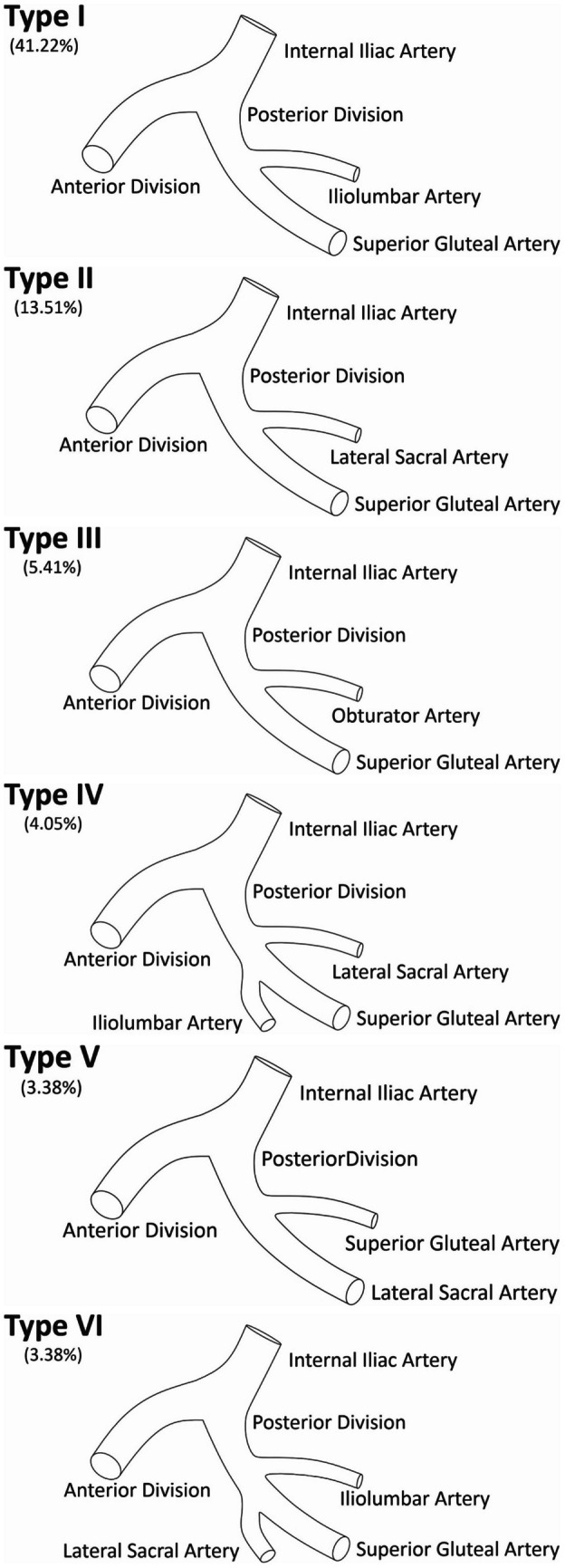
Illustration of the six most common types of the posterior division of the internal iliac artery established in this study

Initially, 21 different arrangements of branches of the PDIIA were noted. The authors decided to present only those that were prevalent in at least 3.00% of the studied cases. Finally, six branching patterns of the PDIIA were established. In the most prevalent type (41.22%), the first branch of the PDIIA is the iliolumbar artery, followed by the superior gluteal artery. Complete data regarding the said types can be found in Table 2 and Fig. 2.

**Table 2:** Branching patterns of arteries originating directly from the posterior division of the internal iliac artery

Category	Overall		Left		Right	
	*N*	Percentage	*N*	Percentage	*N*	Percentage
Type 1	61	41.22	27	36.70	34	46.55
1. Iliolumbar artery						
2. Superior gluteal artery						
Type 2	20	13.51	10	13.98	10	13.79
1. Lateral sacral Artery						
2. Superior gluteal artery						
Type 3	8	5.41	3	3.50	5	6.90
1. Obturator artery						
2. Superior gluteal artery						
Type 4	6	4.05	5	6.99	1	1.72
1. Lateral sacral artery						
2. Iliolumbar artery						
3. Superior gluteal artery						
Type 5	5	3.38	3	4.11	2	2.70
1. Superior gluteal artery						
2. Lateral sacral artery						
Type 6	5	3.38	4	5.24	1	1.72
1. Iliolumbar artery						
2. Lateral sacral artery						
3. Superior gluteal artery						
Other variations (< 3.00%)	42	28.38	22	29.64	20	27.03

### Measurements

The median diameter of the PDIIA at its origin was found to be 6.66 mm (LQ = 6.05; HQ = 7.15). The median cross-sectional area of the PDIIA at its origin was set to be 34.59 mm^2^ (LQ = 27.87; HQ = 39.70). The median number of branches that depart from the PDIIA was 3.00 (minimum = 1.00; maximum = 4.00). The median distance from the origin of the internal iliac artery to the bifurcation into posterior and anterior division was found to be 42.48 mm (LQ = 37.17; HQ = 52.81). Detailed results in all the studied categories can be found in Table 3.

**Table 3:** Results of the measurements

Category	Median	LQ	HQ	Minimum	Maximum	Mean	SD
PDIIA diameter at its Oorigin (mm)	6.66	6.05	7.15	4.87	9.49	6.69	0.93
PDIIA cross-sectional area at its origin (mm^2^)	34.59	27.87	39.70	18.35	69.13	35.14	10.00
PDIIA angle of departure (°)	63.60	43.21	83.57	8.30	128.34	64.29	27.42
Number of branches departing from the PDIIA	3.00	3.00	3.00	1.00	4.00	2.87	0.46
Distance from the origin of the internal iliac artery to the bifurcation into the posterior and anterior division (mm)	42.48	37.17	52.81	20.27	84.94	45.16	11.37
Iliolumbar artery diameter at its origin (mm)	3.25	2.69	3.70	1.39	5.26	3.21	0.70
Iliolumbar artery cross-sectional area at its origin (mm^2^)	7.36	5.16	10.16	1.29	20.91	7.89	3.60
Iliolumbar artery angle of departure (°)	101.13	81.71	116.23	24.45	153.24	98.12	25.21
Lateral sacral artery diameter at its origin (mm)	2.68	2.32	3.18	1.38	9.66	2.81	0.87
Lateral sacral artery cross-sectional area at its origin (mm^2^)	5.13	3.74	7.07	1.34	15.00	5.61	2.51
Lateral sacral artery angle of departure (°)	106.82	86.34	119.98	19.78	158.00	103.14	26.72
Superior gluteal artery diameter at its origin (mm)	6.17	5.71	6.91	2.87	10.99	6.34	1.05
Superior gluteal artery cross-sectional area at its origin (mm^2^)	29.22	25.07	36.74	6.31	93.07	31.76	11.13
Superior gluteal artery angle of departure (°)	94.42	67.77	116.35	5.93	156.62	89.08	33.14
Accessory iliolumbar artery diameter at its origin (mm)	2.59	2.47	2.71	2.47	2.71	2.59	0.17
Accessory iliolumbar artery cross-sectional area at its origin (mm^2^)	5.17	4.65	5.69	4.65	5.69	5.17	0.74
Accessory iliolumbar artery angle of departure (°)	107.03	103.33	110.72	103.33	110.72	107.03	5.23
Accessory lateral sacral artery diameter at its origin (mm)	3.20	2.48	3.91	2.48	3.91	3.20	1.01
Accessory lateral sacral artery cross-sectional area at its origin (mm^2^)	7.54	4.44	10.64	4.44	10.64	7.54	4.38
Accessory lateral sacral artery angle of departure (°)	95.76	91.00	100.51	91.00	100.51	95.76	6.72
Distance between the origin of the PDIIA to the origin of the iliolumbar artery (mm)	9.63	4.56	16.93	0.00	32.95	11.26	8.41
Distance between the origin of the PDIIA and the origin of the lateral sacral artery (mm)	11.88	7.26	18.70	0.00	45.87	13.78	8.91
Distance between the origin of the PDIIA and the origin of the superior gluteal artery (mm)	10.02	5.18	22.32	0.00	84.97	17.47	18.39
Distance between the origin of the iliolumbar artery and the origin of the lateral sacral artery (mm)	13.61	6.50	29.90	0.00	69.38	17.92	14.49
Distance between the origin of the iliolumbar artery and the origin of the superior gluteal artery (mm)	13.26	0.00	36.63	0.00	71.27	19.61	20.66
Distance between the origin of the lateral sacral artery and the origin of the superior gluteal artery (mm)	10.36	3.44	25.37	0.00	65.00	15.90	15.97
Distance between PDIIA and the anterior superior iliac spine (straight line; mm)	107.77	102.53	111.69	82.93	124.26	107.20	7.44
Angle between the section designated by the anterior superior iliac spines and the section designated by the anterior superior iliac spine and the origin of the PDIIA (°)	6.50	3.00	11.00	0.00	23.00	7.28	5.42

*LQ* lower quartile, *HQ* higher quartile, *SD* standard deviation, *PDIIA* posterior division of the internal iliac artery

### Sexual Dimorphism

Statistically significant differences (*p* < 0.05) between females and males were found to occur in only six of the studied categories. In five of them, the median values obtained in males were larger. Detailed results regarding sexual dimorphism can be found in Table 4.

**Table 4:** Results of the measurements with respect to the patients’ sex. Only those results in which a statistically significant difference was found are presented

Category	Sex	Median	LQ	HQ	Minimum	Maximum	Mean	SD	*p* value
PDIIA diameter at its origin (mm)	Females	6.44	5.70	7.04	4.87	8.42	6.44	0.84	0.00
	Males	6.90	6.32	7.58	5.55	9.49	7.04	0.94	
PDIIA cross-sectional area at its origin (mm^2^)	Females	31.66	24.93	38.01	18.35	54.78	32.49	8.56	0.00
	Males	36.56	30.78	44.51	23.65	69.13	38.78	10.77	
Superior gluteal artery diameter at its origin (mm)	Females	6.05	5.60	6.71	4.50	8.32	6.19	0.94	0.02
	Males	6.36	5.98	7.05	2.87	10.99	6.55	1.15	
Superior Gluteal artery cross-sectional area at its origin (mm^2^)	Females	28.06	24.12	35.01	14.78	54.14	30.06	9.39	0.03
	Males	31.53	27.39	38.63	6.31	93.07	34.02	12.84	
Distance between PDIIA and the anterior superior iliac spine (straight line; mm)	Females	105.12	101.37	110.22	82.93	123.94	105.52	7.29	0.00
	Males	110.25	105.29	114.88	88.64	124.26	109.47	7.09	
Angle between the section designated by the anterior superior iliac spines and the section designated by the anterior superior iliac spine and the origin of the PDIIA (°)	Females	9.00	4.00	14.00	0.00	23.00	9.38	5.64	0.00
	Males	4.00	2.00	7.00	0.00	14.00	4.46	3.53	

*LQ* lower quartile, *HQ* higher quartile, *SD* standard deviation, *PDIIA* posterior division of the internal iliac artery

*p* values were established using the Mann–Whitney *U* test

*p* values lesser or equal to 0.05 were considered statistically significant

### Correlations

The data from nine studied categories statistically significantly (*p* < 0.05) correlated with patients’ age. Four of the parameters were found to decrease with age, whereas the remaining five were found to increase with age. Detailed results regarding the correlation analysis can be found in Table 5.

**Table 5:** Correlations between the measured parameters and patient’s age. Only those results in which a statistically significant difference was found are presented

Category	Spearman’s R patients’ age	*p* value
PDIIA diameter at its origin (mm)	0.32	0.00
PDIIA cross-sectional area at its origin (mm^2^)	0.31	0.00
PDIIA angle of departure (°)	0.21	0.02
Iliolumbar artery diameter at its origin (mm)	−0.25	0.00
Iliolumbar artery cross-sectional area at its origin (mm^2^)	−0.24	0.00
Lateral sacral artery diameter at its origin (mm)	−0.24	0.01
Superior gluteal artery diameter at its origin (mm)	0.35	0.00
Superior gluteal artery cross-sectional area at its origin (mm^2^)	0.34	0.00
Angle between the section designated by the anterior superior iliac spines and the section designated by the anterior superior iliac spine and the origin of the PDIIA (°)	−0.25	0.00

*PDIIA* posterior division of the internal iliac artery

*p* values less than or equal to 0.05 were considered statistically significant

Spearman's R correlation test was used in this statistical analysis

### Side Differences

No statistically significant differences (*p* < 0.05) were established in a comparison between the right and left sides of the patients.

## Discussion

The PDIIA typically gives rise to three primary branches: the iliolumbar artery, the lateral sacral artery, and the superior gluteal artery. However, several meta-analyses exploring the anatomical characteristics of arteries originating from the anterior division of the internal iliac artery have revealed that these vessels can also emerge from the posterior division, albeit with variable frequencies [4, 5]. Notably, there is a noticeably low number of studies specifically examining the anatomical features of the PDIIA, including its branching pattern and pelvic location. Most previously published research on pelvic arterial anatomy has concentrated on the entire internal iliac system [6–12], frequently employing traditional classifications such as the Adachi system developed in 1928 [13]. The Adachi classification, with its five types and eight groups, posits that the umbilical artery is the primary stem of the internal iliac artery, with other pelvic arteries (e.g., superior gluteal, internal pudendal) being its branches from an embryological perspective [11]. However, this classification has undergone multiple revisions over the last century owing to oversimplification and practical limitations, failing to showcase all the internal iliac artery branches such as the uterine or iliolumbar arteries. Subsequent classifications have emerged to better elucidate iliac artery branching patterns and their clinical implications. The most recent classification, proposed by Balcerzak et al. [14], emphasizes practical aspects and visual representation of the main branches arising from the internal iliac artery, including the presence or absence of the umbilical artery. Yet, this new system focuses less on detailed branching patterns without descriptions of each individual branch.

Despite numerous existing classifications demonstrating the general anatomy of the internal iliac artery, there remains a scarcity of data specifically detailing the branching pattern and anatomical features of the PDIIA. Thus, our study introduces a novel classification of PDIIA branching patterns and their prevalence. Our classification identifies six types: type 1 (41.22%) features the iliolumbar artery as the first branch and the superior gluteal artery as the terminal branch. Conversely, type 2 (13.51%) begins with the lateral sacral artery, followed by the superior gluteal artery. Type 3 (5.41%) shows the obturator artery followed by the superior gluteal artery. Types 4 and 6 represent the PDIIA, giving rise to its three traditional branches in different orders. Type 4 (4.05%) showcases the lateral sacral artery as its first branch, and type 6 (3.38%) demonstrates the iliolumbar artery as its first branch. In both types, the superior gluteal artery is the terminating branch. Finally, type 5 consists of the superior gluteal artery as the first branch and the lateral sacral artery as the terminating branch (3.38%). Other observed variations (*n* = 42) were less prevalent (< 3.00%) and thus not included in our classification system.

Few studies have thoroughly examined the anatomy of the PDIIA. Bleich et al. [15] conducted a cadaveric study focusing on the general anatomy and clinical implications of the PDIIA, particularly in managing obstetrical hemorrhage. They found that in 62.30% of pelvic halves, posterior division arteries originated from a common trunk, whereas the remainder arose independently from the PDIIA. Additionally, the iliolumbar artery was the most frequently observed first branch (28.3%). Our imaging-based analysis reveals that the predominant initial branch of the PDIIA is the iliolumbar artery (57.76%), whereas the superior gluteal artery is commonly identified as the terminating branch (90.52%). We also examined the prevalence of each branch originating directly from the PDIIA, aligning closely with descriptions in major anatomical references. Notably, the superior gluteal artery (77.03%), iliolumbar artery (53.38%), and lateral sacral artery (32.43%) were the most frequent direct branches. Interestingly, the obturator artery was identified as a direct branch of the PDIIA in a notable percentage of cases (8.11%), contrasting with its conventional origin from the anterior division, a finding supported by other studies [6, 16]. Furthermore, branches that are traditionally known as branches of the anterior division were found to originate from the PDIIA in 13.51% of the individuals. Overall, our findings underscore the considerable variability in pelvic arterial anatomy.

A comprehensive understanding of the PDIIA and its branches is critical for various procedures directly involving these structures. The ligation of the internal iliac artery remains a viable option for managing pelvic hemorrhage [3], particularly in obstetrical and gynecological scenarios such as placenta previa, uterine rupture, and hemorrhage related to advanced pelvic cancers [17–19]. However, this procedure presents challenges, notably the importance of avoiding ligation proximal to the branches of the PDIIA to prevent complications such as buttock claudication and necrosis, as highlighted in previous studies [15, 20, 21]. Accurate identification of the anterior and posterior divisions of the internal iliac artery is crucial for successful ligation. Our study contributes valuable data on the arterial anatomy of the iliac system, including the median distance from the origin of the internal iliac artery to its bifurcation into the anterior and posterior divisions (42.48 mm). We also present an anatomical map highlighting the most frequent locations of this arterial bifurcation. The lower number of branches typically associated with the PDIIA (median = 3) compared with the more complex branching pattern of the anterior division aids in distinguishing between these vessels, facilitating precise arterial ligation.

Injury to specific branches of the PDIIA, such as the iliolumbar artery, can result in severe hemorrhage [22]. Therefore, accurate localization of PDIIA branches is crucial for effectively managing and controlling potential bleeding. Our study provides reliable data that can assist surgeons in this endeavor. By tracing from the origin of the PDIIA, our study outlines median distances to key branches such as the iliolumbar (9.63 mm), lateral sacral (11.88 mm), and superior gluteal arteries (10.02 mm), aiding in precise branch identification and subsequent hemorrhage control if required. It is important to note that the morphometric properties of the analyzed vessel may showcase age-related differences. This is shown in Table 5, where all measured parameters increased with older age. This includes the origin of the branches of the PDIIA, data that are relevant for interventional radiologists who control bleeding with endovascular treatments, such as embolization [23].

The variable vasculature of the pelvis presents significant risks for surgeons performing gynecological procedures, particularly vaginal sacrospinous fixation for apical support [24]. The sacrospinous ligament is in close proximity to critical structures such as the internal pudendal and inferior gluteal vessels, the sciatic nerve, and branches of the sacral nerve plexus as they exit the pelvis via the greater sciatic foramen [25, 26]. Notably, the anatomy of the inferior gluteal artery is highly variable, and its proximity to the sacrospinous ligament places it at heightened risk during vaginal sacrospinous fixation [27]. In our cohort, the inferior gluteal artery originated from the PDIIA in 5.41% of cases. This aberrant origin can lead to a more tortuous and unpredictable course of the artery, increasing the likelihood of injury during surgical procedures. Such injury could result in severe hemorrhagic complications [28]. Additionally, our study highlights that branches typically arising from the anterior division often originate from the PDIIA, further underscoring the complexity and variability of pelvic vasculature.

The present study undoubtedly has several limitations. Radiological imaging is limited to evaluating arteries that are hemodynamically functional. As a result, this introduces a significant bias when assessing anatomical variations of the PDIIA and other vascular structures. Furthermore, the studied sample consisted solely of results from the Polish (white) population. Despite its limitations, this study was aimed at providing comprehensive morphological and anatomical data on the PDIIA, aligning with the principles of evidence-based anatomy [29, 30].

## Conclusion

Our study highlights the critical significance of understanding the PDIIA and its branches in surgical interventions aimed at managing pelvic hemorrhage. The present study provides valuable insights into the precise localization and characteristics of the PDIIA and its branches, which are essential for surgical procedures targeting specific vessels to effectively control bleeding. Owing to the high level of variability of the branching pattern of the PDIIA, a novel classification system consisting of six types was created. The most common direct branches of the posterior division were the iliolumbar artery and the superior gluteal artery. Moreover, the most common first and terminating branches of the PDIIA were found to be the iliolumbar artery and superior gluteal artery respectively. It is hoped that the results of the present study may aid physicians in minimizing hemorrhagic complications associated with various pelvic surgeries.

## Data Availability

The data presented in this study are available on request from the corresponding author.

## Declarations

### Bioethical Committee

The research protocol was submitted for evaluation and approved by the Jagiellonian University Bioethical Committee, Cracow, Poland (1072.6120.254.2022).
